# Screening fungal strains isolated from a limestone cave on their ability to grow and precipitate CaCO _3_ in an environment relevant to concrete

**DOI:** 10.17912/micropub.biology.000764

**Published:** 2023-05-03

**Authors:** Aurélie Van Wylick, Simon Vandersanden, Karl Jonckheere, Hubert Rahier, Lars De Laet, Eveline Peeters

**Affiliations:** 1 Research Group of Microbiology, Department of Bioengineering Sciences, Vrije Universiteit Brussel, Brussels, Brussels Capital, Belgium; 2 Research Group of Physical Chemistry and Polymer Science, Department of Materials and Chemistry, Vrije Universiteit Brussel, Brussels, Brussels Capital, Belgium; 3 Research Group of Architectural Engineering, Department of Architectural Engineering, Vrije Universiteit Brussel, Brussels, Brussels Capital, Belgium; 4 Current Address: Centre for Environmental Sciences, Hasselt University, Hasselt, Flanders, Belgium

## Abstract

Fungi-mediated self-healing concrete is a novel approach that promotes the precipitation of calcium carbonate (CaCO _3_ ) on fungal hyphae to heal the cracks in concrete. In this study, we set out to explore the potential of fungal species isolated from a limestone cave by investigating their ability to precipitate CaCO _3_ and to survive and grow in conditions relevant to concrete. Isolated strains belonging to the genera *Botryotrichum sp.* , *Trichoderma sp.* and *Mortierella sp.* proved to be promising candidates for fungi-mediated self-healing concrete attributed to their growth properties and CaCO _3_ precipitation capabilities in the presence of cement.

**Figure 1. Screening limestone cave fungi as potential candidates for self-healing concrete: graphical abstract and experimental results:**
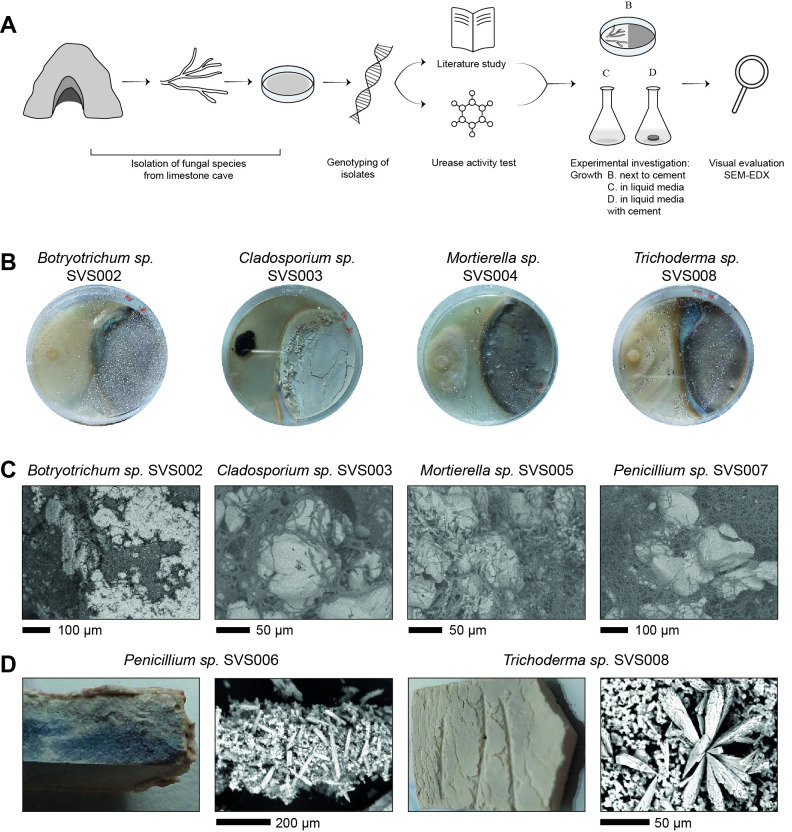
( **A** ) Graphical abstract of the research: first, filamentous fungal strains were isolated from a limestone cave using different growth media, the isolates were then genotypically characterized followed by a literature study and experimental urease activity test. Based on these results, a selection of strains was examined in conditions relevant to concrete to assess their potential as self-healing agents. The selected strains were assessed on their ability to grow next to cement and their ability to precipitate CaCO _3_ in liquid media with and without the addition of cement. The experimental results were analysed both visually and by means of scanning electron microscopy (SEM). ( **B** ) Growth of the isolates next to cement by Botryotrichum sp. SVS002, Cladosporium sp. SVS003, Mortierella sp. SVS004 and Trichoderma sp. SVS008. ( **C** ) SEM images (JSM-IT300 InTouchScope™) of fungal hyphae and CaCO _3_ crystals in modified AP1 medium amended with 50 mM CaCl _2_ : *Botryotrichum* sp. SVS002, *Cladosporium* sp. SVS003, *Mortierella* sp. SVS005 and *Penicillium* sp. SVS007. ( **D** ) Pictures and SEM images (Phenom Desktop) of fungal biomass with precipitated CaCO _3_ layers on top of a cement slab: formed by *Penicillium* sp. SVS006 and by *Trichoderma* sp. SVS008.

## Description

Concrete is a construction material susceptible to crack formation, leading to reinforcement corrosion and material degradation. Calcite, here further referred to as calcium carbonate (CaCO _3_ ), is autogenously present in concrete and is therefore a compatible self-healing product (Seifan et al., 2016) . Microbially induced calcite precipitation (MICP) is a biomineralization technique that enables self-healing of cracks thereby extending the lifespan of concrete (Castro-Alonso et al., 2019; De Muynck et al., 2010; Fahimizadeh et al., 2020; Wiktor and Jonkers, 2016; Wu et al., 2012) . Various microbial species have a native ability to precipitate CaCO _3_ on their cell surfaces through biomineralization (Fahimizadeh et al., 2020; Van Wylick et al., 2021) . The ureolytic MICP pathway involves the formation of calcium precipitates due to the catalysis of urea to ammonium, caused by the production of the urease enzyme, in turn increasing the local pH of the medium (Menon et al., 2019) . Carbonates that are produced from the urea then react with calcium included in the medium to form CaCO _3_ (Fahimizadeh et al., 2020) .

The use of bacteria for MICP-mediated self-healing of concrete has already been extensively investigated: it is used in practice and enables healing of cracks of up to 1 mm (Basilisk, n.d.). However, research on the use of fungi as self-healing agents has only recently emerged (Luo et al., 2018; Menon et al., 2019; Zhang et al., 2021; Zhao et al., 2022) . Filamentous fungi form a promising alternative due to an extensive network-like hyphal growth, thereby providing more abundant biomineral nucleation sites as compared to bacterial self-healing agents. This could potentially aid in a faster healing of larger-sized cracks and in a better protective performance of the self-healed concrete. Also, certain fungal species are adapted to growing in harsh environmental conditions, ensuring biological activity in the concrete environment, the latter being characterized by a high alkalinity and by nutritional scarcity. An additional advantage is offered by native biosorption and bioremediation capabilities of fungi, for example with regards to heavy metals that are also relevant for concrete applications.

Despite the enormous phylogenetic diversity of filamentous fungi, only a limited number of species has been tested for their applicability in self-healing concrete applications (Van Wylick et al., 2021) . These were selected either based on extensive prior biological knowledge and genetic tools, such as the model species *Aspergillus nidulans* , *Trichoderma reesei* and *Neurospora crassa* (Luo et al., 2018; Menon et al., 2019; Zhao et al., 2022) , or upon isolation from an anthropogenic concrete environment (Zhao et al., 2022). In this work, we have selected a limestone cave as a natural habitat for fungi that have a propensity of performing well in self-healing concrete. Indeed, the alkaline, dry and oligotrophic conditions of a limestone cave closely resemble the conditions in a concrete environment. A graphical abstract is provided to clarify the general concept and methodology of this study ( **Figure 1A** ).

First, fungal strains were isolated from a limestone cave by using six types of growth media ( **Table 1** ) to ensure that every strain would sufficiently grow on at least one medium. These growth media differed in their nutrient concentrations (nutrient-rich *versus* nutrient-poor) and pH value (acidic *versus* alkaline). The isolated strains were phylogenetically classified on a genus level using sequencing of an internal transcribed spacer (ITS) region in ribosomal DNA, which is a fast evolving genomic region that varies among species and genera (White et al., 1990) . Based on this molecular identification, it was found that the isolates belong to thirteen different genera/species: *Akanthomyces sp.* , *Aspergillus sp.* , *Botryotrichum sp.* , *Chaetomium sp.* , *Cladosporium sp.* , *Geomyces sp.* , *Ilyonectria robusta* , *Mortierella sp* ., *Mucor racemosus* , *Trichoderma sp.* , *Penicillium sp.* , *Pseudogymnoascus sp* . and *Verticillium sp* . The urease activity of all species was assessed with Modified Christensen’s agar medium. Together with a literature study, with non-pathogenicity as main criterium, a selection of isolates was made for a further experimental study ( **Table 2** ). As a positive control, these experiments were also performed for *Neurospora crassa* , a fungal model species for which a potential for self-healing concrete had already been demonstrated (Li et al, 2014; Zhao et al, 2022) .

The selected isolates were studied for their capability to grow adjacent to one-week-cured cement paste, which indicates their tolerance to grow in conditions relevant for a concrete environment ( **Figure 1B** , **Table 2** , **Extended data 1** ). The strains *Trichoderma sp.* SVS008, *Botryotrichum sp.* SVS002 and *Mortierella sp.* SVS005 were able to grow very well closely to a one-week-cured cement paste slab. *Mortierella sp.* SVS004 showed good growth as well, although this was localized less close to the cement paste. The growth of *Penicillium sp.* SVS006 was limited yet went in the direction of the cement layer. Finally, the strains *Aspergillus sp* . SVS001, *Cladosporium sp* . SVS003 and *Penicillium sp.* SVS007 grew poorly, and their growth was restricted to the opposite side of the cement layer.

Next, the selected isolates were tested for their ability to precipitate CaCO _3_ in liquid medium in absence and presence of a cement paste piece. CaCO _3_ precipitation was witnessed for all the strains ( **Table 2** , **Figure 1C -D** ), including the positive control ( **Extended data file 1** ). Scanning electron microscopy (SEM) showed that the CaCO _3_ crystal morphology varied among the different species ( **Figure 1C** ). These differences might be linked to the varying pH values of the medium after the two-week growth period, which ranged between 7.33 and 9.18, with the starting pH being 5.5 ( **Table 2** ). Another possible explanation for these differences in crystal morphologies are morphological or physiological differences between the selected fungi. Upon incubating the selected strains and *N. crassa* in liquid medium in the presence of a cement paste piece, a layer of fungal biomass with precipitated CaCO _3_ was formed on top of the cement through biomineralization ( **Table 2** , **Figure 1D** , **Extended data file 1** ). This indicates the ability of the fungi to become attached to the cement. The corresponding SEM images show how the fungal hyphae are encrusted by the CaCO _3_ crystals, as they serve as a nucleation site for the precipitation ( **Figure 1D** ).

**Conclusion**

Our work has demonstrated that strains *Botryotrichum sp.* SVS002 *, Trichoderma sp* . SVS008 and *Mortierella sp.* SVS004 and SVS005 showed the highest potential for the application, as they proved to be tolerant to environments relevant to concrete and were able to precipitate CaCO _3_ on cement paste. Apart from these experimental results, a literature study confirmed that these species possess the characteristics to make them suitable candidates for the self-healing concrete applications. *Botryotrichum sp.* is phylogenetically closely related to *Chaetomium sp.,* of which some species are adapted to live in alkaline conditions with a pH value of up to 12 which is atypical for fungi and beneficial for the application (Chinnarajan et al., 2010; Ravindran and Naveenan, 2011) . *Trichoderma sp* . has already been studied in the field of self-healing concrete (Luo et al., 2018; Menon et al., 2019) , giving it a head start in future research. The strain isolated in this study may have advantages over the already studied *Trichoderma reesei* given that it was isolated from an alkaline and oligotrophic environment. Finally, *Mortierella sp.* also performed well in the functional tests and has been previously proposed as a good candidate for self-healing concrete (Van Wylick et al., 2021) thanks to its presence and activity in moonmilk (Park et al., 2020) . Overall, our findings suggest that if microorganisms are used in industrial settings, candidate species selection should take place in environments where the species have evolved to have the desirable characteristics for the application.

## Methods

***Sampling and isolation of fungal strains***

Samples were collected in a limestone cave in Riemst, Belgium (50° 47′ 25″ N, 5° 37′ 02″ E) by scraping fresh mycelium using sterilized tweezers and transporting them in sterile tubes on ice. Subsequently, samples were used to inoculate the following solid growth media: Modified Melin-Norkrans (MMN) medium, modified Fries medium, modified Dichloran Chloramphenicol Rose Bengal (DCRB) medium, potato dextrose agar (PDA) medium, malt extract agar (MEA) medium and MEA+0.5% Ca(OH) _2_ medium ( **Table 1** ). Plates were incubated in the dark at room temperature and observed every 24 hours. At the first signs of visible growth, the mycelial colony was recovered and used to re-inoculate a fresh MEA plate. In this way, all isolates were obtained over a period of maximally 28 days. To preserve the isolated fungal strains, mycelium discs with a 5-mm diameter were taken from the colonies and transferred into sterile 2 mL tubes with 1 mL distilled water and placed in a -20°C freezer. *Neurospora crassa* (WT FGSC #2489, Fungal Genetics Stock Centre (FGSC), Kansas, U.S.A.) was included as a positive control in the study. This strain was maintained on MEA medium at 30°C with 12-hour light/dark cycles.

***Molecular identification of fungal isolates***

Fungal biomass was taken from cultures that were grown on MEA plates with cellophane films to avoid agar contamination and crushed using a mortar and pestle and liquid nitrogen. Next, the material was dissolved in Tris-EDTA (TE) buffer and DNA was extracted using a chloroform/phenol extraction procedure at -20°C. DNA was quantified using a NanoDrop One spectrophotometer (Thermo Fisher Scientific) and amplified by polymerase chain reaction (PCR). Each 25-µl PCR reaction mixture was composed of 12.5 µl KAPA HiFi master mix (Roche Diagnostics), 10-20 ng template DNA and 30 pmol of each primer. The primers used were ITS1 (5’-TCCGTAGGTGAACCTGCGG-3’) and ITS4 (5’-TCCTCCGCTTATTGATATGC-3’), which amplify a section of the large subunit ribosomal DNA of approximately 600 base pairs that is often used for phylogenetic determination (Bellemain et al., 2010) . PCR conditions were as follows: 3 minutes at 95°C, followed by 40 cycles of 2 minutes at 98°C, 15 seconds at 58°C and 50 seconds at 72°C and finally followed by 5 minutes at 72°C. PCR samples were analyzed by agarose gel electrophoresis, followed by DNA gel purification using the Wizard SV Gel and PCR Clean-Up System (Promega) according to manufacturer’s instructions and finally dissolving the purified PCR fragment in 30 µl of RNase-free H _2_ O. Next, the ITS fragments were Sanger-sequenced (Eurofins Genomics) and the obtained sequences were compared to the National Biotechnology Information Centre (NCBI) database using BLAST to determine fungal species that have similar DNA sequences.

***Urease activity test***

Modified Christensen’s agar medium ( **Table 1** ) was used to assess the urease enzymatic activity of the isolates. Thymol blue was used, which has a colour transition from yellow to blue at pH values 8.0-9.6. The pH of the medium increases due to the production of ammonium (NH _4_^+^ ) when urea is broken down by the fungi, causing the change in colour (Martuscelli et al., 2020) . However, weak urease activity might remain undetected as a smaller increase in pH will not lead to a colour transition.

***Growth of isolated fungal strains in the presence of cement***

To test microbial growth in conditions relevant to a concrete environment, an experimental setup was designed employing cement paste. This paste was made by mixing 7 g of sterilized cement (CEM III:B 42.5N N-LH/SR LA, Holcim) with sterilized water (cement/water ratio of 0.5). Next, the paste was poured into a 90-mm sized Petri dish in such a way that half of the plate was covered. After a curing period of seven days at room temperature the other half of the Petri dish was filled with MEA ( **Table 1** ). The part with the growth medium was inoculated with a 5-mm diameter mycelial disc of each isolated fungal strain. The Petri dishes were incubated at 30°C for 21 days. Afterwards the growing behaviour was visually assessed based on the ability of the fungi to grow towards to the concrete.

***CaCO _3_ precipitation test***

For all tested isolates, a spore solution was prepared by scraping spores from solid culture medium and dissolving these in sterilized water. The CaCO _3_ precipitation experiment was adopted from (Li et al., 2014) with minor modifications. Liquid modified AP1 medium ( **Table 1)** was prepared, transferred to a 100 mL Erlenmeyer flask and inoculated by adding 1 mL spore solution. Cultures were incubated in a shaker-incubator at 120 rpm and 30°C for two weeks. Additionally, the influence of a seven-day cured cement paste piece of approximately 1.30 g present in the liquid medium was explored.

***Scanning electron microscopy and energy dispersive X-ray spectroscopy***

Scanning electron microscopy (SEM) combined with energy dispersive X-ray spectroscopy (EDX) were used to visualize the morphology of the precipitated CaCO _3_ crystals and to characterize their composition. The fungal biomass was first retrieved from the Erlenmeyer flasks after two weeks of incubation. The biomass was killed with 70% ethanol and dried in the oven. Once completely dry the samples were crushed to a powder, mounted on the sample carriers with carbon tape and then analysed with SEM-EDX. Two different SEM-EDX devices were used: Phenom Desktop and JSM-IT300 InTouchScope™ with accelerating voltages of respectively 10 kV and 15 kV.

**Tables**

**Table 1** . **Different growth media used in this work.**

**Table:** 

**Medium**	**Recipe**	**pH**	**Reference**
Media used for isolation of environmental fungi			
MMN	10 g.L ^-1^ glucose, 3 g.L ^-1^ malt extract agar, 0.05 g.L ^-1^ CaCl _2_ , 0.025 g.L ^-1^ NaCl, 0.25 g.L ^-1^ (NH _4_ ) _2_ PO _4_ , 0.50 g.L ^-1^ KH _2_ PO _4_ , 0.15 g.L ^-1^ MgSO _4_ *7H _2_ O, 100 µg.L ^-1^ thiamine HCl, 25 mg.L ^-1^ FeCl _3_	5.8	(Rossi and Oliveira, 2011)
Modified Fries	1.0 g.L ^-1^ (NH _4_ ) _2_ .tartate, 0.1 g.L ^-1^ MgSO _4_ .7H _2_ O, 30.0 mg.L ^-1^ KH _2_ PO _4,_ 26 mg.L ^-1^ CaCl _2_ *2H _2_ O, 20 mg.L ^-1^ NaCl, 100 mg.L ^-1^ KCl, 15 mg.L ^-1^ H _3_ BO _3_ , 5.75 mg.L ^-1^ ZnSO _4_ *7H _2_ O, 1.25 mg.L ^-1^ CuSO _4_ *5H _2_ O, 8.5 mg.L ^-1^ MnSO _4_ *H _2_ O, 0.2 mg (NH _4_ ) _6_ Mo _7_ O _24_ *4H _2_ O, 20 mg.L ^-1^ FeCl _3_ *6H _2_ O, 6 g.L ^-1^ D-glucose, 10 g.L ^-1^ agar, 10 ml.L ^-1^ vitamin solution with composition: 1 g.L ^-1^ myo-inositol, 10 mg.L ^-1^ thiamine.HCl, 2.5 mg.L ^-1^ biotin, 10 mg.L ^-1^ pyrodoxine, 10 mg.L ^-1^ riboflavin, 10 mg.L ^-1^ nicotinamide, 10 mg.L ^-1^ p-aminobenzoic acid, 10 mg.L ^-1^ Ca-pantothenate	4.8	ATCC
Modified DCBR	5.0 g.L ^-1^ peptone, 10.0 g.L ^-1^ glucose, 1.0 g.L ^-1^ KH _2_ PO _4_ , 0.002 g.L ^-1^ Mg(SO _4_ ), 15.0 g.L ^-1^ agar	5.6	Oxoid, Thermo Fisher Scientific
PDA	200.0 g.L ^-1^ potatoes (infusion form), 20.0 g.L ^-1^ dextrose, 15.0 g.L ^-1^ agar	5.6	(Aryal, 2015)
MEA	20.0 g.L ^-1^ malt extract, 10.0 g.L ^-1^ agar	5.5	This work
MEA + 0.5% Ca(OH) _2_	20.0 g.L ^-1^ malt extract, 5.0 g.L ^-1^ Ca(OH) _2_ , 10.0 g.L ^-1^ agar	9.7	This work
Urease activity test			
Modified Christensen agar	1 g.L ^-1^ glucose, 1 g.L ^-1^ peptone, 12 mg.L ^-1^ thymol blue, 2 g.L ^-1^ disodium phosphate, 2 g.L ^-1^ sodium chloride, 20 g.L ^-1^ urea ,15 g.L ^-1^ agar	6.8	(Christensen, 1946)
CaCO **_3_** precipitation media			
Modified AP1	111 mM D-glucose, 330 mM urea, 4 mM K _2_ HPO _4_ *3H _2_ O, 0.8 mM MgSO _4_ *7H _2_ O, 2mM NaCl, 9 x 10 ^-3^ mM FeCl _3_ *6H _2_ O, 50 mM CaCl _2_ Trace metals: 1.4 x 10 ^-2^ mM ZnSO _4_ *7 H _2_ O, 1.8 x10 ^-2^ mM MnSO _4_ *4H _2_ O, 1.6 x 10 ^-3^ mM CuSO _4_ *5H _2_ O	5.5	(Li et al., 2014)

**Table 2. Overview of strains that were isolated from the limestone cave and selected for further study.** Experimental results of the strains’ urease activity, growth next to cement, CaCO _3_ precipitation in liquid medium with corresponding pH values and growth on cement in liquid medium are given. Relevant literature for the application is provided as well. Strains appearing twice have been sampled at a different location in the cave. *Urease activity was not detected but could however be present at low levels.

**Table:** 

**Isolated strain**	**Urease positive**	**Growth next to cement**	** CaCO _3_ precipitation in liquid medium **	**End pH liquid medium**	**Growth on cement in liquid medium**	**Relevant literature**
*Aspergillus sp.* SVS001	Yes	Poor	Yes	9.18	Yes	(Gharieb et al., 1998; Kristensen et al., 2021; Menon et al., 2019)
*Botryotrichum sp.* SVS002	Yes	Favorable	Yes	9.13	Yes	(Trovão and Portugal, 2021)
*Cladosporium sp.* SVS003	Yes	Poor	Yes	8.77	Yes	(Martuscelli et al., 2020)
*Mortierella sp.* SVS004	No ^*^	Good	Yes	8.41	Yes	(Cacchio et al., 2014; Park et al., 2020; Xie et al., 2017)
*Mortierella sp.* SVS005	Yes	Favorable	Yes	8.13	Yes	
*Penicillium sp.* SVS006	Yes	Limited	Yes	7.41	Yes	(Ertit Taştan, 2017; Kristensen et al., 2021)
*Penicillium sp.* SVS007	Yes	Poor	Yes	8.39	Yes	
*Trichoderma sp.* SVS008	Yes	Favorable	Yes	7.33	Yes	(Khushnood et al., 2022; Luo et al., 2018)

## Extended Data

Description: Growth and CaCO3 properties of Neurospora crassa. Experiment B: growth next to cement; experiment C: CaCO3 precipitation; experiment D: the addition of cement to the growth medium.. Resource Type: Image. DOI: 10.22002/6y698-v7831
